# Status of active trachoma infection among school children who live in villages of open field defecation: a comparative cross-sectional study

**DOI:** 10.1186/s12889-021-12106-8

**Published:** 2021-11-09

**Authors:** Demoze Delelegn, Alemu Tolcha, Hunachew Beyene, Berhan Tsegaye

**Affiliations:** 1 Department of Ophthalmology, College of Medicine and Health Science, Hawassa University, Hawassa, Ethiopia; 2 Department of Enviromental Health Science, College of Medicine and Health Science, Hawassa University, Hawassa, Ethiopia; 3 Department of Environmental Health, College of Medicine and Health Science, Hawassa University, Hawassa, Ethiopia; 4 Department of Midwifery, College of Medicine and Health Science, Hawassa University, Hawassa, Ethiopia

**Keywords:** Open field defecation, Active trachoma, School children, Sidama

## Abstract

**Background:**

Although many efforts are made by different stakeholders, magnitude of active trachoma remains high among children in Ethiopia. Open field defecation was found to be the main source of active trachoma. However, comparative information on the effect of open field defecation and non-open field defecation on active trachoma is scarce in Ethiopia.

**Methods:**

Comparative community based cross-sectional study was conducted from June 1–30, 2019 in Boricha and Dale districts to assess prevalence of active trachoma among primary school children. We have selected four primary schools purposively from two districts in Sidama. Study participants were selected by using simpe random sampling method. Data were collected through face to face interview, direct observation and ophthalmic examination. Logistic regression analysis was conducted to assess factors associated with active trachoma infection among primary school children. Adjusted Odds Ratios with 95% confidence interval and *p*-value less than 0.05 were computed to determine the level of significance.

**Result:**

From the total of 746 study participants, only 701 study participants gave full response for interview questions making a response rate of 94%. The overall prevalence of active trachoma infection was 17.5% (95% CI, 14.1–20.8) among primary school students. Specifically, prevalence of active trachoma infection was 67.5% among children who lived in open field defecation villages, but it was 88.5% among school children who live in Non-ODF Kebeles. Factors like: Living in open field defecation Kebeles (AOR = 2.52, 95% CI, 1.5–4.1), having ocular discharge (AOR = 5.715, 95% CI, 3.4–9.4), having nasal discharge (AOR = 1.9, 95% CI, 1.06–3.39), and fly on the face (AOR = 6.47, 95% CI, 3.36–12.44) of children were positively associated with active trachoma infection. However, finger cleanness (AOR = 0.43, 95% CI, 0.21–0.9) was protective factor against active trachoma infection in this study.

**Conclusion:**

Significant variation in prevalence of active trachoma infection among school children between open filed and non-open field defecation Kebeles was observed. Surprisingly, the prevalence in open field defecation was significantly lower than non-open field defecation. Hence, this indicates active trachoma infection highly depends on the hand hygiene than environmental sanitation. Educational campaign of hand hygiene should be enhanced in the community for school students.

## Background

Globally, active trachoma is predominant cause of preventable blindness. It is mainly caused by bacterium *Chlamydia trachomatis*. Trachoma infection is transmitted from infected to healthy person during contact of infected eye discharge though hands, clothing,fomites, and excreta [1]. Musca sorbens, eye-seeking fly, are the vector for active trachoma infection [2]. In Ethiopia, Bacterium *Chlamydia trachomatis* and *Trachomatous trichiasis* are concentrated in densely populated regions such as: Amhara, Oromia, and Sidama [3].

After the first infection, active trachoma is manifested sequentially in different clinical stages: First, trachoma manifests as a follicular conjunctivitis, with superficial keratitis and corneal vascularization. Second, it progresses to conjunctival scarring and eyelid distortion with repeated infection gradually. Third, cornea can be damaged. Finally, it can result into chronic inflammation and blindness [4, 5]. Specifically, trachomatous inflammation–follicular (TF) presented as the occurrence of five or more small white follicles (each measure ≥0.5 mm diameters) in the central zone of the upper tarsal conjunctiva. Over time, persistent abrasion of cornea by inward folding of eyelashes could lead to cornea opacification and blindness. Finally, repeated infection can lead to *trachomatous trichiasis* [6, 7].

Globally; an estimated 84 million people are suffering from active *trachoma* infection. According to World Health Organization (WHO) estimation, trachoma infection is an endemic disease in 56 Middle East and Africa countries. The problem can be doubled by lack of extensive resources necessary for prevention of trachoma such as: Access to clean water, sanitation facilities and health resources in developing countries [8]. Trachoma endemic communities are usually dry and dusty areas, mired in poverty, and have poor sanitation [9, 10]. Trachoma is expected to be endemic throughout Ethiopia [11]. According to 2005–2006 national survey report, over 9 million children were affected by active trachoma [12]. Specifically, prevalence of active trachoma is expected to be high in the study area [13].

Children are the most vulnerable groups of the population for trachoma infection [14]. Two-fifth of active trachoma infection is occurred among children of age 1–9 years [3]. Active trachoma infection (trachomatous inflammation follicularis and/or trachomatous inflammation intense) reaches peak at the age of pre-school (4–6 years); however, subsequent scarring and blindness can also be occurred among children [7]. Blindness affect not only individuals but also several poverty reduction strategies [15]. Visual impairment can lead to annual productivity loss of $3–$6 billion in the world. Hence, sustainable development goal cannot be achieved in 2030 [16]. In Ethiopia, trachoma is the country’s second most common cause of blindness next to cataract [17]. Approximately two-fifth of blindness is believed to be avoidable in Ethiopia prevention and early treatment [15]. Currently, despite of significant effort made to eliminate trachoma, trachoma is still a major health crisis in Ethiopia [18].

Multiple factors are assumed to be associated with active trachoma infection [13]. Specially, active trachoma infection among children is caused by presence of flies on the face [19], large family size [20], ocular discharge, nasal discharge, low socioeconomic status, living in higher altitude and unsafe water sources [21]. On the contrary, frequent washing of children’s face, clean environment and hygienic disposal of excrement were found to be preventative factors [22]. Several studies indicate that open field defecation is the main risk factor for trachoma infection [19, 23, 24]. On the contrary, other evidence proved that latrine access was not associated with active trachoma or *C. trachomatis* infection, despite the fact that 60% of individuals do not have access to a latrine. Latrine access does not translate into latrine use [25].

World health organization has targeted trachoma to eliminate trachoma from being as a public health problem by 2020. Therefore, SAFE strategy (surgery for in-turned eyelashes, antibiotics to clear infection, and facial cleanliness and environmental cleanness) was designed and implemented in the last couple of years in the world [25, 26]. The targets for elimination of trachoma in Ethiopia include: Reduce prevalence of Trachomatous inflammation follicular below 5% among children for children aged 1–9 years at the health district level and to minimize trachomatous trichiasis (TT) less than one case per 1000 among the total population at health district level [27]. As a result, policymakers and program designers should know the prevailing problem and factors hindering the elimination of trachoma to design better and logical programs and policy. In this study, we hypothesized that active trachoma should be significantly higher in open field defecation Kebeles than non-open field defecation.

## Methods

### Study design, setting and period

This comparative cross-sectional study was conducted to determine the prevalence and factors associated with active trachoma infection among primary school children. This study was carried out in the technology village of Hawassa University, Southern Ethiopia. It is located in Sidama regional state 310 km from the capital city of the Ethiopia (Addis Ababa). Specifically, the village is found in Boricha and dale districts. It consists 6 districts and 168 Kebeles. An estimated 636, 146 (97%) people live in rural areas. The total health service coverage of the village was 85%. Generally, there are 633 primary schools in Sidama regional state. There were 360,547 (181,543males and 179,004 females) school age children in the region. The common cause of disease and disability in the region is associated with lack of clean drinking water, poor sanitation and low public awareness about environmental health and personal hygiene practices in the region. As of 2010/2011 population estimation report, Boricha and Dale districts had a total of 42 and 39 Kebeles respectively. There were 1 hospital, 17 health centres 74 health posts 7 private medium clinics and 1 non-governmental clinic in both districts. The study was done in primary schools (1–6 grades) of Kebeles; the technology Village of Hawassa University, in Boricha and Dale districts. This study was conducted from June 1–30; 2019.

### Sample size and sampling procedure

The minimum sample size, which can meet the study’s objectives, was determined by balancing of the following components of the study: Power, precision, baseline proportion, quality and cost of the study. The sample size was calculated for both prevalence of active trachoma infection and factors associated with active trachoma infection. Hence, the calculated sample size using variables from the study of blindness, low vision and trachoma in Ethiopia gave the largest sample size of 678 for this study [3]. Furthermore, assuming 10% non-response rate, the final sample size was estimated as 746 for this study.

A total of 4 from 12 Kebeles were selected in the study settings. Two Kebeles were selected from each district (One from ODF and One from Non-ODF). They were selected purposefully considering the following assumptions: Absence of trachoma project in these Kebeles at the time of study period, their representativeness for latrine coverage and water coverage. Regarding about sample Kebeles, Debub Mesenkela (ODF) and Tula (Non-ODF) were selected from Dale district and Yirba Dewoncho (Non-ODF) and Konsore Arkie (ODF) were selected from Boricha district. Study participants were selected using simpe random sampling (lottery) scheme. Updated list (roster) of the students was utilized as the sampling frame in each section. The sample size for each section was allocated was using probability proportional to the total number of student for that section. Revisits of two times were made in case where eligible respondents were not available at the time of the data collection.

### Population

In this study, the source population were all primary school children in both Boricha and Dale districts. Whereas, the study population were primary school students who were selected for this study and presented at time of data collection. Each child was the study unit in this study. School children who were actively learnt for more than 6 months in the selected primary schools were included in the study. Children who were unable to give response at the time of data collection due to severe illness like: Mental illness, unconsciousness and depression were excluded from this study.

### Study variables

Outcome variable was the presence of active trachoma infection. Active trachoma infection is defined as Trachomatous inflammation—follicular or Trachomatous inflammation—intense or Trachomatous scarring among children age 1–9 years. The outcome variable is dichotomized. If active trachoma was proved to exist, it were labelled as “1”; otherwise, “0”. The independent variables were include the socio-demographic characteristics of the children’s parent: Mother’s educational status, father’s educational status, ethnicity, family income, religion, and child care taking practice, family size, and parent’s marital status and sharing room with animals. Moreover, behavioural characteristics of study subjects included: Ocular discharge, nasal discharge, flies on face, finger cleanness, open defecation field and status of cloth cleanness. The age of the study participants were taken from the sampling frame. Wealth index was calculated using principal components analysis [28]. It was formed in three stages containing many indicators [29].

### Data collection

In this study, various types of data collection method were used. Data was collected through face to face interview, and ophthalmic examination. We have adopted questionnaire from national survey on blindness, low vision and trachoma in Ethiopia. Furthermore, we have adapted standard checklist from Thylefors, B simple system for assessment of trachoma and its complications [6, 11, 30]. The tool was first developed in English and translated into Sidama afoo (local language) and then back to English to keep its consistency. Comparison was done between two versions to assess inconsistent and inaccurate data. School principals arranged appropriate time and place for data collection. Data were collected by eight qualified and trained BSC degree ophthalmic professionals for ophtalaminc examination. Furthermore, there were four BSC nurses who collect socio-demographic and hygiene-related information. Two masters of public health (MPH) professions supervised data collection process of this study. They had previous experience in supervising data collection. Finally, any inconsistent and inaccurate data were corrected accordingly.

### Data analysis

Analysis was done using SPSS version 24. Descriptive analysis was made using percentage, mean, standard deviations for variables included in the study. Bivariate and multivariate logistic regression analyses were done to identify the association between the independent and outcome variables. Before the actual logistic regression analysis was done, the necessary assumption of logistic regression model was checked. Frequencies were first determined followed by cross tabulations to compare frequencies. At bi-variate level, analysis was made by the chi square (X^2^) test for categorical variables. The association between dependent and independent variables was measured by means of odds ratio for which 95% confidence interval was calculated. Variables which showed a statistically significant association (*p* < 0.25) at bivariate logistic regression analysis was further fitted into multivariate logistic regression analysis. Finally, variables which have *P*-value of less than 0.05 were considered as significantly associated factors of ‘trachoma infection’ in the multivariable logistic regression analysis. Adjusted odds ratios with the 95% Confidence Intervals (CI) were reported. Hosmer-Lemeshow test of goodness of fit was used to check model fitness.

### Data quality control

Data collectors and supervisors were given three-day training before the start of actual data collection time. The training was focused on the aim of the study, sampling procedure and data collection technique. Qualified and re-oriented ophthalmic professionals were recruited. Before utilization in the field, the data collection tool for ophtalaminc examination was calibrated accurately. All data were registered immediately after collection, and checked for completeness. Pre-test was conducted on 37(5%) of study participants in Wondogent district which is out of the current study settings.

### Ethics approval and consent to participate

Ethical clearance was obtained from Institutional Review Board (IRB) of College of Medicine and Health Science of Hawassa University. Furthermore, regional and district health offices were communicated through official letter of permission. Relevant school directors and teachers were well informed about the scope and objective of the study. The study protocol was clarified for parents /legal guardians/ of the study subjects and informed written consent was taken from parents or legal guardians of the study participants. Children were treated with 1% TTC eye ointment or referred if they were infected with active trachoma.

## Results

From a total number of 748 study participants, only 701(94%) study participants gave full response in the this study. The socio-demographic characteristic of the study population is shown in Table 1. Regarding the fathers of children in the study, only 14.2% from ODF and 8.5% from non-ODF Kebeles of could read and write. Nearly one third (31.6%) of children lived in the middle wealth quintile household. Three hundred seventy three (53.2%) of parents of the study subjects cared for their children.

**Table 1:** Socio-demographic characteristics of the children’s parent in Dale and Boricha districts, Sidama Ethiopia, in 2019(*n* = 701)

Characteristics	Category	ODF	Non- ODF	Total
Mothers educational status	Can nor read and write	145 (20.7%)	145 (20.7%)	290 (41.37)
	Read and write	118 (16.8%)	40 (5.7%)	158 (22.54)
	Primary	135 (19.3%)	5 (0.7%)	140 (19.97)
	Secondary	26 (3.7%)	7 (1%)	33 (4.71)
	College and above	76 (10.8%)	4 (0.6%)	80 (11.41)
Fathers educational status	Cannot read and write	299 (42.7%)	561 (80%)	160 (22.82)
	Read and write	122 (17.4%)	81 (11.6%)	203 (28.96)
	Primary education	99 (14.1%)	3 (0.4%)	102 (14.55)
	Secondary education	69 (9.9%)	14 (2%)	83 (11.84)
	College and above	111 (15.9%)	42 (6%)	153 (21.83)
Religion	Protestant	94 (13,4%)	17 (2.4%)	111 (15.83)
	Orthodox	51 (7.2%)	196 (28%)	247 (35.24)
	Muslim	42 (6%)	252 (36%)	294 (41.94)
	Others	7 (1%)	42 (6%)	49 (6.99)
Ethnicity	Oromo	56 (8)%	42 (6%)	98 (13.98)
	Amhara	26 (18%)	84 (12%)	110 (15.69)
	Sidama	144 (20.5%)	220 (31.3%)	364 (51.93)
	Others	70 (10%)	59 (8.4%)	129 (18.40)
Economic status	Poorest	75 (10.6%)	96 (13.6%)	171 (24.39)
	Poor	47 (7%)	68 (9.7%)	116 (16.55)
	Middle	168 (24%)	54 (7.%)	222 (31.67)
	Rich	25 (3.6%)	84 (12%)	109 (15.55)
	Richest	56 (8%)	28 (3.9%)	84 (11.98)
Child care taking	Care taker	289 (41.2%)	84 (12%)	373 (53.21)
	Non-care taker	266 (38%)	62 (8.8%)	328 (46.7)9
Family size	< 4 persons	369 (52.6%)	40 (5.7%)	409 (58.35)
	> 4 persons	232 (33%)	60 (8.5%)	292 (41.65)
Marital status	Married	148 (21.1%)	322 (46%)	470 (67.05)
	Divorced	84 (12%)	77 (10.9%)	161 (22.97)
	Widowed	14 (2%)	42 (6%)	56 (7.99)
	Single	14 (2%)	49 (7%)	63 (8.99)
Sharing room with animals	Yes	290 (41.3%)	299 (42.7%)	589 (84.02)
	No	65 (9.3%)	47 (6.7%)	112 (15.98)

Accordingly, one out of two study participants were selected from open field defecation Kebeles and the remaining study subjects were selected from non-open field defecation Kebeles. Based on the districts, more than half (55.9%) of study participants were selected from Boricha district; whereas, the remaining 45.1% were selected from Dale district. More than four-fifth of the study participants had ocular discharge at the time of data collection. Nighty eight (14.4%) of the study participants had nasal discharge at the time of data collection. More than half (54.4%) of the study participants had no finger cleanness during inspection at data collection. Males accounted more than half (51.4%) of study subjects in the current study. Most of the study participants were in the age range of 5–9 years (82.7%), the mean age of 8.8 + 1.67 years. Regarding about cloth hygiene, more than half (53.5%) of the study participants had no cloth cleanness. Only 1 out of 10 (8.3%) study participants had fly on their face, while most (91.7%) of children did not have fly on their face during data collection time. According to Table 2 report, we found that the overall prevalence of active trachoma infection was 17.5% among primary school students in the study area. Specifically, the prevalence of trachoma infection was 67.5% in the ODF villages, while the prevalence of active trachoma infection was 32.5% in non-ODF Kebeles.

**Table 2:** Behavioral characteristics of study participants in Boricha and Dale districts, Sidama Ethiopia, in 2019 (*n* = 701)

Variables	Number of students	Percent
**Kebeles**		
Debub Mesenkela	152	21.7
Tula	157	22.4
KA	201	28.7
Yirba dewoncho	191	27.2
**Woreda**		
Boricha	392	55.9
Dale	309	44.1
**Age**		
5–9 years	580	82.7
10–14 years	131	18.7
15 years and above	90	12.8
**Sex**		
Male	360	51.4
Female	341	48.6
**Ocular discharge**		
Yes	135	19.3
No	566	80.7
**Nasal discharge**		
Yes	101	14.4
No	600	85.6
**Fly on face**		
Yes	58	8.3
No	643	91.7
**Finger cleanliness**		
Yes	320	45.6
No	381	54.4
**Cloth cleanliness**		
Yes	326	46.5
No	375	53.5
**Trachoma infection**		
Yes	123	17.5
No	578	82.5
**Type of trachoma**		
TF	71	57.7
TI	37	30.1
TS	15	12.2
**ODF status**		
ODF	353	50.4
Non-ODF	348	49.6

Key: *TF* Trachomatous inflammation Foliculris, *TI* Trachomatous Inflammation Intense, Trachomatous Scarring, *ODF* Open defecation field

### Factors associated with active trachoma infection

According to Table 3 reports, living in ODF Kebeles, being female, having ocular discharge, having nasal discharge and having flies on the face, cloth cleanness and finger cleanness were factors associated with active trachoma infection in chi-square statistics (X^2^)). Moreover, all variables which show significant association in chi-square distribution statistics were further analysed in the binary logistic regression analysis. Hence, living in ODF Kebeles, being female, having ocular discharge, having nasal discharge and having flies on the face were factors positively associated with active trachoma. On the contrary, cloth cleanness and finger cleanness were factors negatively associated with active trachoma in the current study.

**Table 3:** Hygiene and sanitation status of the study subjects in Boricha and Dale districts, Sidama Ethiopia, 2019 (*n* = 701)

Variables	ODF Status		
	ODF N(%)	Non ODF N(%)	X^**2**^; ***p*** value
**Trachoma infection**			
Yes	83 (67.5%)	40 (32.5%)	17.496, 0.000
No	270 (46.7%)	308 (53.3%)	
**Ocular discharge**			
Yes	72 (53.3%)	63 (46.7%)	0.593; 0.441
No	281 (49.6%)	285 (50.4%)	
**Nasal discharge**			
Yes	52 (51.5%)	49 (48.5%)	0.06; 0.8
No	301 (50.2%)	299 (49.8%)	
**Fly on face**			
Yes	32 (55.2%)	26 (44.8%)	0.587; 0.444
No	321 (49.9%)	322 (50.1%)	
**Finger cleanliness**			
Yes	150 (46.9%)	170 (53.1%)	2.855; .091
No	203 (53.3%)	178 (46.7%)	
**cloth cleanliness**			
Yes	155 (47.5%)	171 (52.5%)	1.926; 0.165
No	198 (52.8%)	177 (47.2%)	

Based on the report of Table 4, except cloth cleanness, factors which significant association in bivariate logistic regression analysis model also significantly associated in the multivariate logistic regression analysis model. On the other hand, family size, child caring practice, sharing of rooms with animals, educational status and wealth quintile did not show significantly association in this study. Primary school children in the ODF schools were 2.5 times more likely to be infected with active trachoma infection than their counterparts (AOR = 2.52, 95% CI, 1.5–4.1, *p* < 0.000). The odd of getting active trachoma among female primary school children were 1.63 times higher than males (AOR = 1.6; 95% CI, 1.2–2.6, *p* < 0.046). The chance of children who had ocular discharge was 5.7 times more than their counterparts (AOR = 5.71, 95% CI, 3.5–9.4, *p* < 0.000). The likelihood of children with nasal discharge were 1.8 times more likely than their counterparts (AOR = 1.89, 95% CI, 1.06–3.39, *p* < 0.031). The odd of children with flies on their face were 6.5 times more likely than their counterparts (AOR = 6.5, 95% CI, 3.3, 12.4, *p* < 0.000). Furthermore, those children with clean finger were experienced 57% points less likely to be infected with active trachoma infection than their counterparts (AOR = 0.43, 95% CI, 0.21, 0.86, *p* < 0.018).

**Table 4:** Factors associated with active trachoma infection from backward stepwise (Wald) logistic regression analysis among study participants in Boricha and Dale districts, Sidama Ethiopia, in 2019 (*n* = 701)

Hygiene conditions	Trachoma infection			
	Yes N (%)	No N (%)	AOR (95% CI)	***P***- value
**ODF status**				
ODF	270 (76.5%)	83 (23.5%)	2.52 (1.5–4.1)	0.000
Non-ODF	308 (88.5%)	40 (11.5%)	1	
**Sex of students**				
Male	305 (84.7%)	55 (15.3%)	1	
Female	273 (80.1%)	68 (19.9%)	1.631 (1.009–2.638)	0.046
**Ocular discharge**				
Yes	66 (48.9%)	69 (51.1%)	5.715 (3.468–9.418)	0.000
No	57 (10.1%)	509 (89.9%)	1	
**Nasal discharge**				
Yes	42 (41.6%)	59 (58.4%)	1.898 (1.060–3.399)	0.031
No	81 (13.5%)	519 (86.5%)	1	
**Fly on face**				
Yes	35 (60.3%)	23 (39.7%)	6.472 (3.366–12.444)	0.000
No	88 (13.7%)	555 (86.3%)	1	
**Finger cleanliness**				
Yes	27 (8.4%)	293 (91.6%)	.431(.215–.863)	0.018
No	96 (25.2%)	285 (74.8%)	1	
**Cloth cleanliness**				
Yes	28 (8.6%)	298 (91.4%)	.647(.326–1.285)	0.214
No	95 (25.3%)	280 (74.7%)	1	

## Discussion

We anticipated high prevalence of active trachoma in open field defecation Kebeles among children of 1–9 years in this study. However, it has demonstrated that active trachoma was found to be high in non-open field defecation Kebeles. The prevalence of active trachoma among school children was 76.5% in ODF Kebeles; the prevalence of active trachoma was 88.5% among school children who live in Non-ODF Kebeles. This proportion was statistically significant (x^2^ = 17.5, *p*-value < 0.000). These findings indicate that 12% of excess active trachoma was observed among school children in non-ODF Kebeles. This mainly indicates that presence of latrine only does not give guarantee for prevention of active trachoma elimination rather the health behaviour related with hygiene determine the prevalence of active trachoma. From this, we can understand effective utilization of latrine, hand hygiene after utilization of latrine and increase awareness about active trachoma are mandatory apart from transforming ODF Kebeles to non ODF status. The overall prevalence of active trachoma infection was 17.5% (95% CI, 16.1–18.8) among primary school students aged 1–9 years. Specifically, prevalence of trachoma infection was 55.9 and 44.1% in Boricha and Dale district respectively. These findings are reduced almost by half when compared with prevalence of active trachoma in Southern Nation Nationalities Regional State (33.2%). Furthermore, these findings were also lower than age adjusted prevalence of South region (25.9%) [3]. The possible rational for this difference might be due to lack of awareness about personal hygiene and sanitation. Besides, lack of water and hygiene materials in this study might result into higher trachoma infection. Compared to prevalence of active trachoma in 2014, prevalence of active trachoma was higher in 2019 in the study area [31]. This might indicate that despite progress in prevention of active trachoma in the region, little effort was made to prevent active trachoma in the study area in the last couple of years. Ethiopian federal ministry of health, and other stakeholders have identified various type of strategy to control and eradicate active trachoma like: Hygiene and promotion, improved access to clean water, and proper sanitation for disposal of human waste as a priority intervention for trachoma infection and other wash related disease through the health extension program and adoption of community-led total Sanitation [32]. The current study showed that prevalence of active trachoma inflammation follicular (TF) was found to be 55.7%. The current finding is more than the WHO criteria for elimination of trachoma as public health problem i.e. TF < 5% in children aged from 1 to 9 years (WHO) [33]. This might be due to absence of universal supplementation of antibiotics, facial cleanliness and environmental cleanness strategy for trachoma elimination. Previous evidence recommend A, F and E components of the SAFE (Surgery, Antibiotic, Facial cleanliness and Environmental improvement) strategy for trachoma elimination for settings with prevalence of active trachoma infection more than 5% [34]. Primary school children in ODF Kebeles were two and half times more likely prone for active trachoma infection than their counterparts. This finding is in line with previous study conducted in Southern parts of Ethiopia [31, 35]. The possible explanation could be the following: Firstly, active trachoma infection might be due lack of personal hygiene (finger cleanness, facial cleanness and environment hygiene) more than latrine utilization. This indicates that the mere presence of latrine could not guarantee the prevention of active trachoma infection unless appropriate hygiene practices are not performed after latrine utilization [36]. Secondly, community led total sanitation campaigns are practiced by ODF Kebeles more than non-ODF Kebeles. Finally, health education program might be strengthened by community health workers about personal hygiene, environmental sanitation and disease prevention in ODF Kebeles than their counterparts. Similar studies also approved this fact that health education intervention statistically significant reduction in the prevalence of active trachoma [22]. Finally, in these populations children aged 1–9 years are often discouraged from using latrines due to caregiver concerns about their safety when using latrines.

The odd of getting active trachoma infection among primary school children who had flies on their face is higher than their counterparts. This finding is consistent with finding of similar study carried out in East Nigeria [19, 37–39]. The possible rational might be that school children with flies on their face did not wash their face frequently due to lack of water source. Previous evidence indicate that improving access to safe water sources and better hygiene practices can reduce trachoma morbidity nearly by one third [40]. As previous evidence proved that frequent washing of children, clean environment and hygienic disposal of excretion were valuable preventative factors for active trachoma infection [22].

In this study, females were more affected by active trachoma infection than males. This finding is inconsistent with the previous study done in Guinea [41]. On the contrary, females have low odds of getting trachoma infection in the study conducted in Biogas [42]. The possible explanation might be female children are in close contact with vectors and other exposure variables during home works. In addition, some communities might have sex preference in taking care of children.

The chance of children who had ocular discharge more than their counterparts. This finding is consistent with the previous study. This finding is in line with the previous study [41]. The possible rational might be that children who had ocular discharge were more likely to attract flies on their eye and less likely to wash with clean water and soap. Similar study proved that children who did not wash their face with soap and water were more likely affected by active trachoma infection [43].

Children with nasal discharge were more affected than their counterparts. This finding is similar with the previous study [41]. This might be due to the fact that nasal discharge might create good media for attracting flies on their face causing active trachoma infection. Flies are vectors of chlamydia trichromatic infection [44]. Nasal and ocular discharges might result from the inflammation of active trachoma and cause the face to be classified as unclean [24, 45, 46]. At last, children with clean finger had lower risk of infection than their counterparts [47]. The possible explanation might be due to the fact that children with unclean finger had chlamydia Trachomatous with their finger and subsequent transmission through direct contact at the time of touching of their eyes.

### Limitation of the study

This study had number strengths. First, it yields the most reliable result that was conducted with appropriate design and adequate sample size. Second, the response rate was good which further strength the generalization. Finally, we have used random sampling and appropriate mixed type of data collection to incur data at a maximum effort. On the contrary, this study could address only primary school children as it limits the extent of generalization. The cross sectional nature of the study did not show the cause and effect relationship. Furthermore, as the study was a school based one, other risk factors which might be apparent at household and neighbourhood level were not assessed. Hence, multilevel and follow up studies should be conducted to fill this gap.

## Conclusion

Prevalence of active trachoma is high among school children in open field defecation than school children of non-open field defecation. Furthermore, the prevalence of active trachoma among school children is higher than the national prevalence in both areas. Hence, the government should strength proper environmental sanitation, hand hygiene and proper use of latrine. Campaigns focusing on introducing educational intervention programs in women’s organizations, schools, and communities could help bring behavioural changes among school children, which are the change agents, and lower the burden of trachoma in the study area.

## Data Availability

We have sent all the available data and we do not want to share the raw data as we are doing related study.

## Abbreviations

AOR: Adjusted odds ratio
CI: Confidence interval
OR: Odds ratio
ODF: Open defecation free
HEW: Health extension worker
CLTS: Community led total sanitation
NGO: Non-governmental organization
WHO: World health organization
SNNP: Southern nation nationality and peoples
